# Minimally invasive prosthetic procedures in the rehabilitation of a bulimic patient affected by dental erosion

**DOI:** 10.4317/jced.51732

**Published:** 2015-02-01

**Authors:** Giacomo Derchi, Michele Vano, David Peñarrocha, Antonio Barone, Ugo Covani

**Affiliations:** 1 DDS, MSc, PhD, Clinical and research fellow, Tuscan Stomatologic Institute, Lido di Camaiore, Italy; 2 DDS, MSc, PhD, Assistant Professor, Department of Surgical Pathology, Medicine, Molecular and Critical Area, University of Pisa, Italy; 3 DDS,MSc,PhD, Junior Researcher, Oral Surgery Division Dental Clinic, Faculty of Medicine and Dentistry, University of Valencia, Spain; 4 DMD, Professor, Department of Surgical Pathology, Medicine, Molecular and Critical Area, University of Pisa, Italy

## Abstract

The population affected by dental erosion due to bulimia is generally very young. This population group has a high aesthetic requirement; the dentition in these patients is severely damaged, especially in the anterior maxillary quadrant. In terms of treatment, it is still controversial whether an adhesive rehabilitation is preferable to a longer-lasting but more aggressive conventional treatment, such as full-crown coverage of the majority of teeth. This case report describes the prosthetic rehabilitation of a young female patient previously affected by bulimia nervosa and presenting erosion of the maxillary teeth. The prosthetic rehabilitation was performed through indirect adhesive restorations of the anterior teeth and direct restorations of the posterior teeth. A clinical follow-up after 4 years showed that the occlusion remained satisfactorily restored. Posterior direct composite resin restorations and anterior indirect adhesive composite restorations proved to be an effective time and money-saving procedure to rehabilitate patients affected by dental erosion. Adhesive rehabilitation provides a functional and good aesthetic result while preserving tooth structure.

** Key words:**Bulimia, dental erosion, composite resin, veneers.

## Introduction

Bulimia nervosa is an eating disorder characterised by a disturbed eating behavioural pattern, a pathological control of body weight and a distorted perception of body shape (1). Bulimia affects a wide socio-economic group, irrespective of class (2). It is the most common eating disorder currently treated by psychologists and psychiatrists. The sex ratio is approximately 10 females to 1 male (3). The population affected by dental erosion due to bulimia is generally very young (4). Patients affected by this disease eat large quantities of food in relatively short periods and control their body weight through self-induced vomiting. The systematic regurgitation of gastric contents, having an extremely high acidic pH, causes erosion and demineralization of the enamel, which represents the most typical oral manifestation of eating disorders. This specific type of enamel erosion, determined by self-induced vomiting, is defined as perimylolysis and is characterised by the erosion of enamel on the lingual, occlusal and incisal sides of teeth. This results from the chemical action of gastric acids and the mechanical activation induced by the movements of the tongue (5); the erosion is generally more evident on the palatal surfaces of maxillary anterior teeth, which appear smooth and glossy. The treatment of bulimia nervosa is based on a multidisciplinary approach. Besides medical treatment, the dental clinician can contribute to the rehabilitation of bulimic patients by re-establishing the masticatory function and the aesthetical appearance of the eroded teeth. According to the available literature, the recommended therapy comprises both extensive elective root canal treatment and full-crown coverage of the majority of teeth (6). However, this approach, due to the very young age of the population affected by this disease, can be considered too aggressive. Recently a more conservative approach to treat severe dental erosion has been described thanks to the use of adhesive techniques (7,8). It is still, nonetheless, controversial whether an adhesive rehabilitation is preferable to a longer-lasting but more aggressive conventional treatment. In this clinical report, an alternative approach was presented for the prosthetic rehabilitation of a young female patient previously affected by bulimia nervosa, who presented erosion of maxillary teeth.

## Case Report

**Figure 1:**
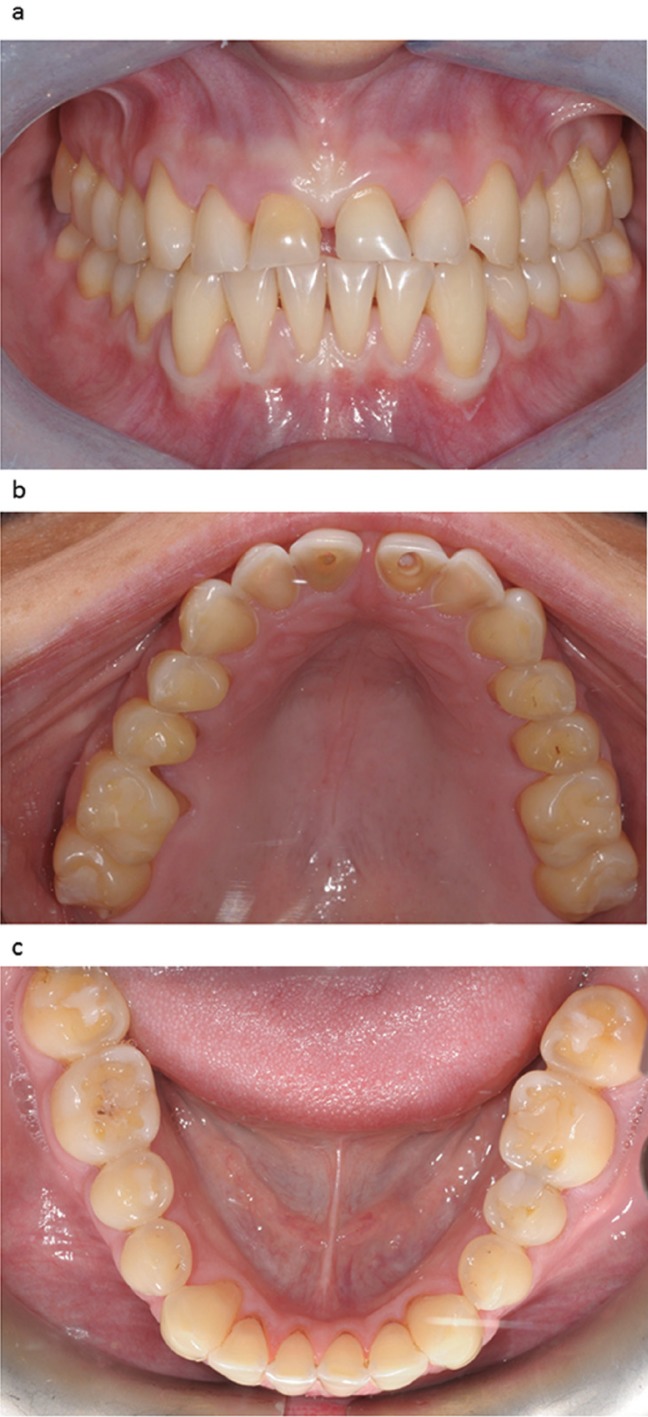
a) Frontal view showing the fractured incisal edges of the central incisors. b) Occlusal view showing severe erosion of the maxillary teeth. Note the loss of vitality of the two central incisors. c) Lower arch: moderate signs of erosion especially on the occlusal surfaces.

**Figure 2:**
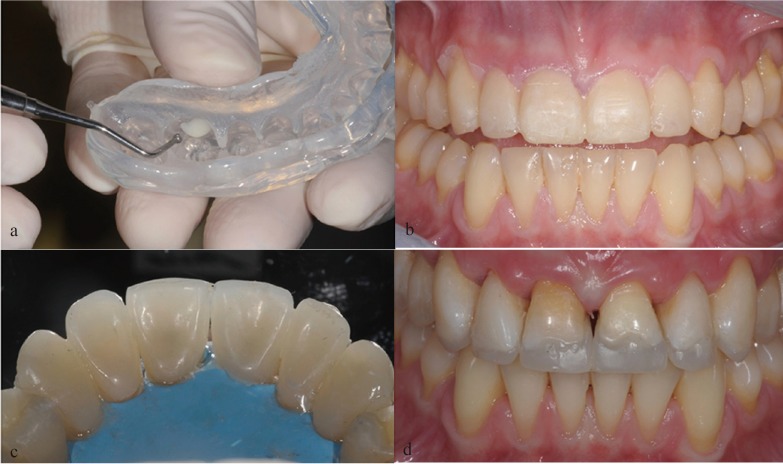
a) Translucent silicone key loaded with composite to fabricate the posterior restorations. b) Positioning of the six maxillary anterior teeth in place to check if its aesthetic appearance was very pleasing for the patient. c) Occlusal view of the palatal composite veneers for the anterior maxillary teeth. No root canal retreatment was necessary for the two central incisors and the endodontic access was filled with composite before taking the final impression for the palatal veneers. d) Frontal view of the bonded palatal composite veneers for the six anterior teeth.

A 22-year-old female patient who had previously been affected by bulimia nervosa, presented erosion of the maxillary teeth, which was particularly evident on the palatal side (Fig. 1 a,b). According to the Basic Erosive Wear Examination (BEWE) (9), the sum of the scores of the maxillary sextants was 19 with a risk level classified as high. In the BEWE the most severely affected surface in each sextant is recorded with a four level score and the cumulative score classified and matched to risk levels which guide the management of the condition. The sum of the scores of the mandibular sextants was 3 with a risk level classified as low. According to the anterior clinical erosive classification (ACE) (8), the patient was considered ACE class IV regarding laterals and canines since the palatal dentin was largely exposed and the loss of length of the clinical crowns was more than 2 mm, while the facial enamel was still preserved. The central incisors were classified ACE class VI because of the loss of tooth vitality. In addition the patient presented generalized erosion on the maxillary premolars and molars on the cervical third of the palatal side. The erosion was extended to the occlusal surfaces of the maxillary premolars and molars but only on the palatal cusps, so that the vertical dimension of occlusion was entirely preserved by the vestibular cusps. On the other hand, the mandibular teeth presented only moderate signs of erosion especially at the level of the occlusal surface (Fig. 1c). The observation of minimal erosion to erosive damage of the mandibular teeth in bulimic patients has been described in previous published studies (5). The patient reported that she was unhappy with the appearance of her teeth. The teeth were vital with the exception of the two maxillary central incisors, which had previously received a root-canal treatment. During the first examination, she stressed her desire to have a restoration procedure with a high aesthetic result, without the use of metal. Periodontal screening resulted in no pathological findings; the Periodontal Screening Index (PSI) was 0 in all sextants. The patient’s oral hygiene was good. She had no signs or symptoms of temporomandibular disorders. The patient insisted on having a rapid and effective aesthetic and functional result, but with a limited budget. To enable the clinician and the dental technician to study the clinical situation and to choose the most appropriate treatment option in the first clinical session, intra and extraoral photographs of the patient were taken as well as two polyvinyl siloxane impressions (Flexitime Dynamic Heavy Tray, Flexitime Correct Flow, Heraeus-Kulzer, Hanau, Germany). The occlusion of the patient and the inclination of the occlusal plane were also recorded with a facebow. During the first laboratory diagnostic step, split-cast models were obtained (VertySystem, A.GREE srl, Altavilla Vicentina, Vicenza, Italy; Elite Rock, Zhermack, Badia Polesine, Rovigo, Italy) and articulated on a semi-adjustable articulator by means of the facebow in the maximum intercuspidation position (MIP) (Artex C, AmannGirrbach, Vorarlberg, Austria; Elite Arti, Zhermack). A functional and morphological evaluation of the casts was simultaneously conducted by the clinician and technician. The aim of the technique outlined in this clinical report was to restore a compromised dentition using both direct bonded posterior composite restorations and indirect adhesive techniques for the anterior teeth (palatal and facial composite veneers). To perform the direct posterior restorations, all the teeth with the exception of the second molars were waxed up (7) (Nawax Compact, Yeti Dental, Engen, Germany) (Vertys Heavy Glass 72 Shore, VertySystem, A.GREE srl). A translucent silicone key (Memosil II, Heraeus-Kulzer) was fabricated reproducing the wax-up of the posterior teeth at the increased vertical dimension of occlusion (VDO) (Fig. 2a). The increase of the VDO was determined looking at the height of the intact vestibular cusps.

During the following clinical session, the clinician loaded several times the translucent silicone key with a tooth-colored composite and positioned it in the patient’s mouth. The second maxillary molars were used as stops for the key. Therefore direct composite restorations were fabricated to restore the maxillary first molar and the two premolars (Clearfil Protect Bond and Clearfil Majesty Posterior, Kuraray Medical Inc, Tokyo, Japan). In order to keep the interproximally contact points opened polytetrafluo-roethylene film (Teflon) was used to protect the adjacent teeth. Another translucent silicon key was fabricated for the six maxilla-ry anterior teeth to prepare the mock-up (Vertys Easy Putty 70 Shore and Vertys Precision 56 Shore, VertySystem, A.GREE srl). The clinician loaded the translucent silicone key with a resin for temporary crowns (SINTODENT S.r.l., Roma, Italy). The mock-up helped the clinician to verify that the aesthetic appearance of the final restoration would have met the expectations of the patient (Fig. 2b). In the same appointment the anterior teeth were prepared to receive six palatal composite veneers. The interproximal contacts between the maxillary anterior teeth were slightly opened using thin diamond strips, and the incisal edges were smoothed by removing the unsupported enamel prisms. The palatal dentin was also cleaned with nonfluoridated pumice, and the most superficial layer was removed with a diamond bur. After this minimal preparation of the palatal surfaces an impression was taken (Flexitime, Heraeus-Kulzer, Hanau, Germany) in order to obtain the palatal veneers described in the Three Steps Technique by Vailati and Belser (10) The dentin was sealed with Optibond FL (Kerr). No provisional restorations were placed.

**Figure 3:**
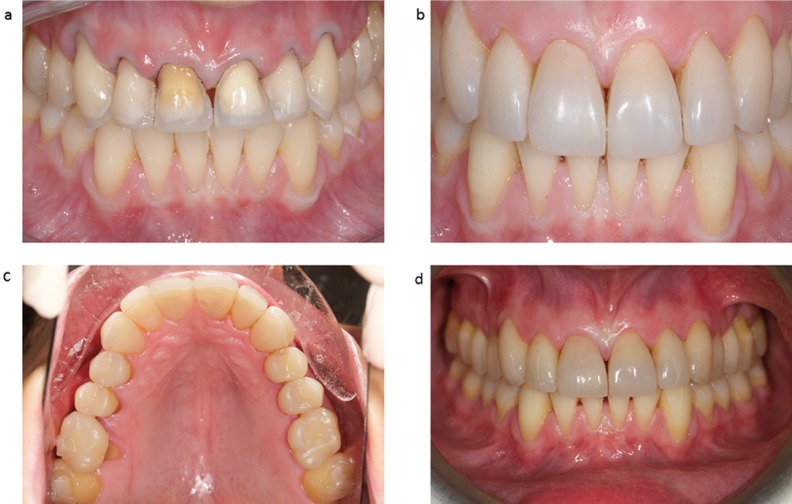
a) Facial veneers preparation, performed immediately after the delivering of the six palatal veneers. b) Final result of the vestibular composite veneer restorations. Each maxillary anterior tooth was restored by means of two composite veneers (palatal and facial), (sandwich approach), to guarantee the maximum preservation of the existing tooth structure. c) Palatal aspect after 4 years of clinical function. d) Frontal view of the vestibular composite veneer restorations after 4 years of clinical function.

Following a similar protocol, previously published by Vailati *et al.* 2012 (7), after one week, the palatal veneers (Estenia C&B, Kuraray Medical Inc) were bonded, one at a time, using rubber-dam isolation (Fig. 2c,d). The palatal sealed dentin was sandblasted (Cojet, 3M ESPE; Seefeld, Germany), the surrounding enamel was etched (37% phosphoric acid), and the adhesive (Clearfil Esthetic Cement Kit, Kuraray Medical Inc) was applied but not cured (7). The composite veneers were also sandblasted (Cojet), cleaned in alcohol, and several coats of silane were applied (Clearfil Esthetic Cement Kit, Kuraray Medical Inc). In the same appointment, after bonding of the palatal veneers, a conservative preparation was performed on the vestibular side of the maxillary incisors and canines (Fig. 3a). To deliver a light chamfer, the six facial veneers were prepared at the cervical level, following the curve of the marginal gingiva, with no need to extend the preparation to the gingival sulcus (in contrast to the crown preparation) (10). After the impression (Flexitime, Heraeus-Kulzer) a provisional was fabricated using a resin for temporary crowns (SINTODENT S.r.l., Roma, Italy) with the same silicon key used for the mock-up. The laboratory step consisted in the realisation of the vestibular composite veneers for the anterior maxillary teeth (Estenia C&B, Kuraray Medical Inc). After one week, in the last clinical session the six veneers were cemented on the anterior teeth following the same procedures described for the palatal veneers (Clearfil Esthetic Cement Kit, Kuraray Medical Inc) (Fig. 3b). The patient came for a first follow-up visit one month later; she was satisfied with the overall treatment. The restorations were well integrated with the other teeth and the soft tissues were very healthy.

Afterwards the patient was scheduled for a 6 month follow-up. During each control a professional air-polishing (11) with glycine powder was performed in order to reduce the plaque accumulation. A clinical follow-up after 4 years showed that the occlusion remained satisfactorily restored thanks to the posterior direct composite resin restorations and anterior indirect composite adhesive restorations (Fig. 3 c,d). Lastly, with regards to the direct composite restorations, no loss, fracture, marginal discoloration or loss of marginal integrity was noted after 4 years of clinical function.

## Discussion

It is well documented that eating disorders have serious consequences on both the physical and emotional health (12). Bulimia nervosa has severe effects on the functionality of the patient’s oral cavity due to self-induced vomiting, leading to severe tooth erosion from stomach acids. The conventional treatment of these patients would require the teeth to be rebuilt using crowns, over-lays and root canal treatment. This would involve a great loss of healthy dental tissue as well as very high expense, which young patients can not always afford. The treatment strategy should be strictly related to the extent of the erosive lesions and, therefore, different therapeutic options are possible. Limited erosion can be treated by placement of direct composite resin restorations, as shown in this clinical report (13,14). Several authors obtained a high success rate in the medium term using the direct technique to reconstruct teeth that had been subjected to erosions or abrasions (7,13,14). Indeed, currently available resin composites are much improved from a mechanical viewpoint and, in addition, offer excellent aesthetic qualities. These materials are, in fact, able to replace missing tissue with very little dental preparation. For erosive lesions with extensive loss of substance, however, composite resin restorations may no longer be an adequate therapeutic option in the long term (15). Prosthetic treatment with a facial and palatal composite (or ceramic) veneer to restore the dentition should then be indicated, as shown in this report. This clinical report shows how to restore a compromised dentition with a time- and money-saving procedure, using direct bonded posterior composite restorations and anterior indirect composite adhesive restorations. A clinical follow-up at 4 years showed that the occlusion remained satisfactorily restored with good aesthetic results. This technique proved to be a valid alternative to traditional treatment for patients affected by severe dental erosion. Finally, thanks to the functional and aesthetic rehabilitation, the patient has overcome her psychological problems to face interpersonal relationships on regaining her self-confidence.
